# Age-Related Changes in Hemispherical Specialization for Attentional Networks

**DOI:** 10.3390/brainsci11091115

**Published:** 2021-08-24

**Authors:** Maria Casagrande, Francesca Agostini, Francesca Favieri, Giuseppe Forte, Jasmine Giovannoli, Angela Guarino, Andrea Marotta, Fabrizio Doricchi, Diana Martella

**Affiliations:** 1 Dipartimento di Psicologia Dinamica, Clinica e Salute, Università di Roma Sapienza, 00185 Roma, Italy; 2 Dipartimento di Psicologia, Università di Roma Sapienza, 00185 Roma, Italy; francesca.agostini@uniroma1.it (F.A.); francesca.favieri@uniorma1.it (F.F.); g.forte@uniroma1.it (G.F.); jasmine.giovannoli@uniroma1.it (J.G.); angela.guarino@uniroma1.it (A.G.); fabrizio.doricchi@uniroma1.it (F.D.); 3 Mind, Brain and Behavior Research Center (CIMCYC), University of Granada, 18011 Granada, Spain; marotta@ugr.es; 4 Department of Experimental Psychology, University of Granada, 53005 Granada, Spain; 5 Facultad de Ciencias Sociales y Humanidades, Instituto de Estudios Sociales y Humanísticos, Universidad Autónoma de Chile, Santiago 7500912, Chile

**Keywords:** attention, attentional networks, alerting, orienting, executive control, aging, lateralization

## Abstract

Many cognitive functions face a decline in the healthy elderly. Within the cognitive domains, both attentional processes and executive functions are impaired with aging. Attention includes three attentional networks, i.e., alerting, orienting, and executive control, showing a hemispheric lateralized pattern in adults. This lateralized pattern could play a role in modulating the efficiency of attentional networks. For these reasons, it could be relevant to analyze the age-related change of the hemispheric specialization of attentional networks. This study aims to clarify this aspect with a lateralized version of the Attentional Network Test for Interaction (ANTI)-Fruit. One hundred seventy-one participants took part in this study. They were divided in three age groups: youth (N = 57; range: 20–30); adults (N = 57; range 31–64), and elderly/older people (N = 57; range: 65–87). The results confirmed the previous outcomes on the efficiency and interactions among attentional networks. Moreover, an age-related generalized slowness was evidenced. These findings also support the hypothesis of a hemispheric asymmetry reduction in elderly/older adults.

## 1. Introduction

Cognitive functions develop during the life span [1,2]. These processes reach their maximum expansion in early adulthood, which results in optimal performance in cognitive tests [1]. When aging advances, there is a physiological decline, which involves physical and cognitive dimensions, interfering with daily routines (e.g., executive functions) [1]. 

Some factors appear to compensate for this physiological decline, as a healthy lifestyle, characterized by adequate physical activity, good eating behaviors [3], absence of alcohol or smoking habits [4], optimal cognitive reserves [5], and optimal levels of blood pressure [6].

Since the aging process is inter-individually different, the functional decline does not happen for all people in the same way. Some results underline that, generally, elderly people preserve some cognitive skills, such as language and crystallized intelligence (i.e., knowledge of general facts). Conversely, other cognitive processes deteriorate, i.e., processing speeds [7], executive functions [8], memory [9], psychomotor skills [10], time perception [11,12], and attention [13]. Specifically, attention plays a fundamental role in cognitive functioning, allowing one to carry out some daily activities that require aware environmental information processing. However, the efficiency of attentional systems depends on the cooperation of different mechanisms (i.e., memory, behavioral and motor responses, and selection of information); for this reason, their functioning and changes during aging are still unclear.

Posner and Petersen [14,15] identified three attentional networks involved in selective attention and associated with different anatomical brain regions (i.e., orienting, alerting, and executive control). The orienting system, located in the parietal cortex, involves direct attention toward space, focusing on specific stimuli (i.e., detecting environmental details) [15]. The executive control, located in the prefrontal cortex and anterior cingulate cortex, allows solving and controlling conflicts between expectations, stimuli, and responses [16]. This network requires perceiving and recognizing stimuli by selecting a single response among many possibilities. Finally, the alerting system, located in the right hemisphere, allows for greater activation and consequently higher responsiveness to stimuli. It is related to arousal systems and sustained attention favoring faster responses to stimuli [15]. 

To assess simultaneously the three different attentional systems suggested by Posner and Petersen’s attention model, several authors adopted the Attention Network Test (ANT) [17]. The ANT combines the spatial cueing task [18] with the flanker task [19], helping in the analysis of the attentional functioning in its complex interactions with specific brain areas [14,15,20]. The use of the ANT has been remarkably successful in studying attentional systems. The basic idea that allows assessing the efficiencies and the interactions of the three attentional systems simultaneously with a simple and short test has been ascertained by many researchers. Some of them have proposed some variants of the original ANT. One well-known variant is the Attentional Network Test for Interaction (ANTI), proposed by Callejas and collaborators [21]. The ANTI, while maintaining the basic structure of the original ANT, introduces some changes in the evaluation of orienting and alerting, which are useful to evaluate the interaction between these systems directly. Invalid spatial cues are included to assess attentional costs and benefits of orienting, and an auditory warning signal is introduced to assess Alerting. In both the ANT and the ANTI, the stimuli used are arrows. According to the hypothesis that the directionality of these stimuli could overstress the executive system, another version of the ANTI with non-directional stimuli (i.e., fruits), i.e., the ANTI-Fruit (ANTI-F) [22], has been proposed as well as a lateralized version of the ANTI-F (LANTI-F; [23]).

The ANT, or some of its variants, has been adopted in several populations (e.g., adults, adolescents, children, and clinical populations) [22,23,24,25,26,27,28,29,30,31]. Considering aging, the ANT has demonstrated that older people are less accurate and generally slower than the younger [32,33,34,35]. 

Analyzing specifically each attentional network in aging, the results are inconsistent. The orienting network seems to be deeply conditioned by the typical slowdown of aging. Accordingly, studies reported slower reaction times and a greater benefit of spatial cues in elderly people. However, once the reaction times are adjusted for the general slowdown, there are no significant differences in the performances between elderly and young people [36,37,38]. Moreover, no difference between the elderly and the young in their abilities to re-orient attention is evidenced by both adopting central versus spatial cues [33,34,35,36,38] and invalid versus valid cues [34]

Considering the executive control, elderly people appear to benefit more from the congruent condition [33,35,36,37,38]. Nevertheless, this effect disappears, when reaction times are corrected for the generalized slowdown.

Finally, concerning the alerting network, most studies have shown no advantage of the warning signal in increasing attentional performances in older people; on the contrary, they would seem disturbed by the acoustic signal [33,35,36,37,38]. However, an aspect which is not properly investigated would be the hemispheric lateralization of attentional networks, which could be affected by the aging process. The first hypothesis about the lateralization of attentional systems has been proposed in studies on neglect. These studies have shown that front-parietal damage in the right hemisphere produces less attention to objects placed in the left visual field [39,40].

On this line, several authors have highlighted that a healthy population experiences a similar pattern (i.e., pseudoneglect) [41] and tends to shift more attention to the left visual field, which involves the activation of the right contralateral hemisphere [42,43]. The preference for the left visual field demonstrating mainly through visual-spatial tasks (e.g., [44,45] would confirm the greater responsibility of the right hemisphere in spatial attention. However, the superiority of the right hemisphere in sustained attention is also evidenced with tasks that do not involve visual stimuli [46,47,48].

To distinctly assess the three attentional networks in each hemisphere, Greene and his colleagues [49] developed a lateralized version of the ANT: The Lateralized Attentional Network Task (LANT). In this task, targets are presented in the right or left visual field, providing a measure of the three networks in each hemisphere (according to Kornad et al. [50]). Additionally, invalid spatial cues are included to assess attentional costs and benefits of orienting, and the warning signal is acoustical, according to the ANTI [21]. 

This first study using the LANT showed that both hemispheres sustain the three attentional networks [50]. Similar results were found by Poynter et al. [51]. Other studies proved the dominance of the right hemisphere in the attentional systems [52,53,54]. These results are consistent with clinical and imaging data showing a clear right hemisphere dominance for attentional functions in both visual and auditory modalities [55,56,57,58,59].

The nature of stimuli can modulate the efficiency of attentional networks and differently involve the hemispheric control. The interaction between executive control and orienting reduces the interference of distractors, when a spatial cue is present [54]. Using the ANTI-F, i.e., adopting non-directional stimuli instead of arrows, it has been demonstrated that directional stimuli can increase the difficulty in resolving the conflict [22]. Furthermore, a lateralized version of the ANT-F (LANT-F) reveals that the three attentional networks interact only when the stimuli are presented in the left visual field (right hemisphere), but not when they are presented in the right visual field (left hemisphere). These findings highlight the preeminent role of the right hemisphere in modulating the best attentional performance when all attentional networks are simultaneously involved. 

All these versions of the ANT, adopting different stimuli or assessing lateralized components of attention furnish new insight into studies of attentional networks in the elderly.

The brain organization changes over time [60,61,62], and these modifications can compromise the efficiency of attentional networks. For this reason, understanding hemispheric lateralization changes in aging appears relevant. 

A recent review by Friedrich and colleagues showed a decrease in the tendency to be more sensitive to stimuli presented in the left visual field in elderly people, confirming brain changes related to aging with decreased right hemisphere specialization for spatial attention [63]. This finding demonstrates a reduction of hemispheric asymmetries in the elderly [64,65] due to a neural reorganization and a decreased right hemisphere dominance for attention in the elderly and older people.

### Aims

This study aimed to explore whether age influences the three attentional networks or specifically affects one of them. Moreover, we intended to verify age effects on the hemispheric asymmetry of the attentional networks. Assuming that the use of non-directional stimuli might be more suitable to assess the efficiency and interactions of the attentional networks, the LANTI-F was used to compare the roles of the right and left hemispheres on the modulation of the attentional networks in youth, adults, and elderly/older people. According to previous results [23], we expected to confirm an asymmetric attentional performance in young and adults; according to recent data [63], this asymmetrical pattern should disappear in elderly/older people.

## 2. Method

### 2.1. Participants

One hundred eighty-three people voluntarily took part in the study. Considering the LANTI-F accuracy, 12 were excluded from the study (3 youth, 3 adults, and 6 elderly/old participants). The final sample was composed by 57 university students (female/male: 39/18; mean age = 23.46 ± 2.08 years; range: 20–30), 57 adults (female/male: 39/18; mean age = 55.26 ± 7.54 years; range: 31–64), and 57 older adults (female/male: 39/18; mean age = 71.82 ± 5.88 years; range: 65–87). Adults and elderly participants were selected according to the classification of the World Health Organization [66] that considers the population aged 65 or over as elderly. Participants reported normal or corrected normal vision, and all of them were naive to the purpose of the experiment. 

### 2.2. Cognitive Status

The Mini-Mental State Examination (MMSE) [67] was used to assess global cognitive status. Participants who had scores of ≤24 on the MMSE corrected by age and educational level were not included in the current study. Then, the participants performed the Raven’s progressive matrices [68] to estimate intelligence quotient (IQ); the raw scores were corrected by age.

### 2.3. Apparatus

Stimuli were programmed and displayed by E-Prime software on a 17” monitor with a screen resolution of 1024 pixels × 768 pixels. Responses were collected through the mouse, and headphones were used to administer the auditory alerting tones. A chinrest was fixed at 56 cm from the monitor to guarantee the appropriate head position of the participants. 

### 2.4. Stimuli

Each trial began with a central cross of 1° (degrees of visual angle). The stimuli consisted of red strawberries and yellow pears, presented on a grey background. They were positioned with the four flankers that overlapped the border of an imaginary semicircle where the target was at the center [23]. This choice was made to ensure that all flankers appeared at the same distance with respect to the central target, avoiding any distortion caused by the reduced efficacy of the leftmost and rightmost flanker stimuli in a row [29]. The flanker could be the same as the target (congruent condition) or different (incongruent condition). The cue was an asterisk of 1°, and it could be presented in the same position as an upcoming target (valid cue condition) and in the opposite location (invalid cue condition), or it could be absent (no-cue condition). The auditory warning stimulus was 2000 Hz and lasted 50 ms.

### 2.5. Procedure 

**Figure 1:**
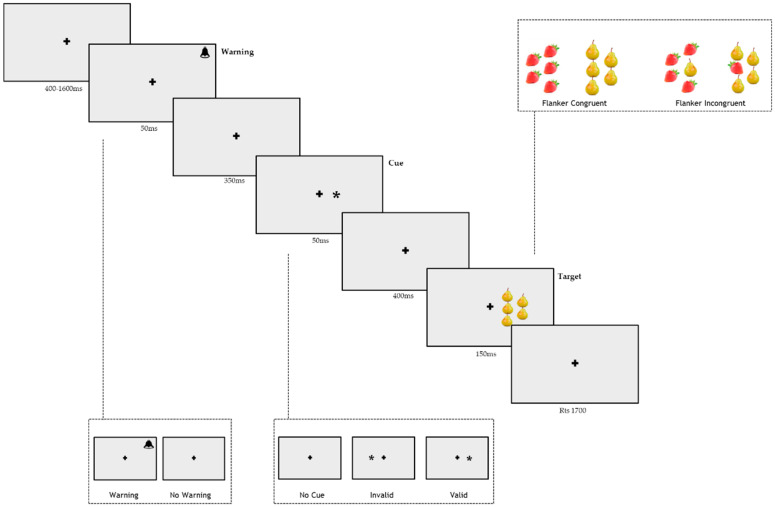
Schematic of trials in the Lateralized Attentional Network Test for Interaction-Fruit (LANTI-F).

Subjects were tested individually in a silent and dimly lit room. Before each trial, the fixation cross was presented for a variable duration (400–1600 ms). The fixation period was followed by a warning stimulus lasting 50 ms in 50% of the trials. After a fixed interstimulus interval (ISI) of 350 ms, a cue of 50 ms was presented. In the valid condition (33% of the trials), an asterisk appeared in the same position of the upcoming target; in the invalid condition (33%), the target appeared in the opposite position than the one signaled by the cue; in the no-cue condition (33%), no orienting stimulus was presented. The flankers were congruent with the target in half of the trials, while the trials were incongruent in the other half. After a stimulus onset asynchrony (SOA) of 400 ms, the target and the flankers were presented for 150 ms in the left or right visual field in order to isolate the information to one hemisphere, 5° from the fixation point. The participants had a limit of 1700 ms to respond. The fixation point was at the center of the screen throughout the trial. The sequence of the events for each trial is shown in Figure 1.

#### 2.5.1. Procedure

The participants performed one practice block of 16 trials, followed by four experimental blocks of 144 trials each. Overall, the participants completed 48 valid trials, 48 invalid trials, and 48 no-cue trials for each flanker and each warning condition. Half of stimuli were presented in the right visual hemifield and half were in the left visual hemifield. Trials were randomly presented within each block. The entire experiment comprised 576 trials for a total duration of around 20 min.

The participants were instructed to fixate the central cross and discriminate the fruit on the semicircle center. Half of the participants responded with the left button of the mouse when the pear was the target and with the right button when the strawberry was the target, while the other half of the participants had the opposite condition.

#### 2.5.2. General Procedure

The experiment was performed following the ethical standards of the Declaration of Helsinki. The Institutional Review Board of the Department of Psychology (protocol number: 0001063) approved the study.

After the participants signed the informed consent, the MMSE and Raven’s progressive matrices were administered. Then participants performed the LANTI-F.

### 2.6. Data Analysis

A power analysis a priori was conducted to define the total sample size, considering a medium effect size. An average sample of 90 (30 for each group) participants was considered adequate for this study.

For the analyses of the LANTI-F, only the RTs of the correct responses ranging between 200 and 1400 ms were considered via automatic e-prime filtering. People who reported accuracies lower than 50% were excluded from the analysis (N = 12; 6.6%). Three young, three adult, and six elderly/older participants were excluded because of their low accuracy rates.

An Age group (Youth, Adults, and Elderly/Older) × Visual Field (Left, Right) × Warning (No-warning and Warning) × Cue (Invalid cue, No-cue, and Valid cue) × Flanker (Congruent and Incongruent) analysis of variance (ANOVA) was conducted on the RTs of the correct responses. 

To estimate the lateralization of each attentional system, an Age group x Visual hemifield ANOVA was conducted on the orienting effect (RTs invalid-cue − RTs valid-cue), the conflict effect (RTs incongruent trials − RTs congruent trials), and the alerting effect (RTs no-warning − RTs warning) [23]. A high score of orienting effect reflects the ability to rapidly orient the attention towards the targets appearing in the cued positions. A smaller conflict effect reflects the ability to inhibit the interfering effect of distractor stimuli (flankers). The alerting effect represents the benefit of alerting on the speed of the response to the target. To limit more challenging conditions that could increase inter-hemispheric activity [69], the alerting effect was calculated, excluding the invalid and incongruent conditions; the orienting effect was computed disregarding the incongruent conditions, and the conflict effect was estimated without considering the invalid trials.

Bonferroni correction was adopted to control multiple comparisons, and a level of significance of adjusted *p*-value of 0.03 was considered to accept the hypothesis. 

A partial correlation analysis (Pearson’s r) was conducted to analyze the relationship between the age and the lateralization of attentional effects in the right and left visual fields, controlling for years of education, MMSE, and IQ. Standard linear regression analyses were used to evaluate whether age influenced attentional effects differently in the right and left visual fields. 

ANOVAs and correlational analyses were conducted with Statistica 10.0, while regression analysis was conducted using SPSS v.25.

## 3. Results

The three groups significantly differ in age (F_2168_ = 1078.4; *p* < 0.0001; η^2^ = 0.93), years of education (F_2168_ = 9.7; *p* < 0.0001; η^2^ = 0.19), and MMSE (F_2168_ = 11.37; *p* < 0.0001; η^2^ = 0.12). The three groups did not differ in IQ (F_2168_ = 3.01; *p* = 0.052; η^2^ = 0.03). However, the planned comparison revealed that Youth’s IQ did not differ from those of Adult (F_1168_ = 3.59; *p* = 0.06; η^2^ = 0.782) and Elderly/Older (F_1168_ = 0.16; *p* = 0.68; η^2^ = 0.14) groups, while the Adult group showed an higher IQ than the Elderly/Older one (F_1168_ = 5.28; *p* < 0.02; η^2^ = 0.84). The means and standard deviations of age, years of education, MMSE, and IQ scores for each group are shown in Table 1. 

### 3.1. LANTI-F

The mean RTs for each experimental condition in both left and right visual fields for the three age groups are shown in Table 2. The overall accuracy for the left visual field was 93.74% (±7.13%), and for the right visual field, it was 93.66% (±7.17%). The percentages of errors in the three groups of participants were 1.3 for Youth, 2 for Adults, and 3 for Elderly/Old.

The ANOVA on RTs showed the significant main effects of *Age groups* (F_2168_ = 62.77; *p* < 0.0001; η^2^ = 0.43), *Warning* (F_1168_ = 289.16; *p* < 0.0001; η^2^ = 0.62), *Cue* (F_1168_ = 144.77; *p* < 0.0001; η^2^ = 0.46), and *Flanker* (F_1168_ = 400.33; *p* < 0.0001; η^2^ = 0.70). 

The participants were faster in the warning than no-warning conditions (F_1168_ = 280.16; *p* < 0.0001; η^2^ = 1.00), and they were faster when the flanker was congruent than incongruent (F_1168_ = 400.33; *p* < 0.0001; η^2^ = 1.00). The participants were also faster in valid trials compared to in invalid (F_1168_ = 239.61; *p* < 0.0001; η^2^ = 0.98) and no-cue (F_1168_ = 45.27; *p* < 0.0001; η^2^ = 0.98) trials.

Furthermore, the Youth group was faster than both Adults (F_4165_ = 45.75; *p* < 0.0001; η2 = 0.29) and Elderly/Older (F_5164_ = 142.89; *p* < 0.0001; η^2^ = 0.56) groups, and the Adults group was faster than Elderly/Older group (F_4165_ = 15.54; *p* < 0.0001; η^2^ = 0.12). 

The main effect of Visual Field was not significant (F_1168_ = 3.18; *p* = 0.08; η^2^ = 0.02); although the means showed a tendency of participants to be faster when stimuli were presented in the left visual field than in the right visual field (753 ± 7.66 ms; 95% CI = 738–768 vs. 757 ± 7.58 ms; 95% CI = 742–772)

**Figure 2:**
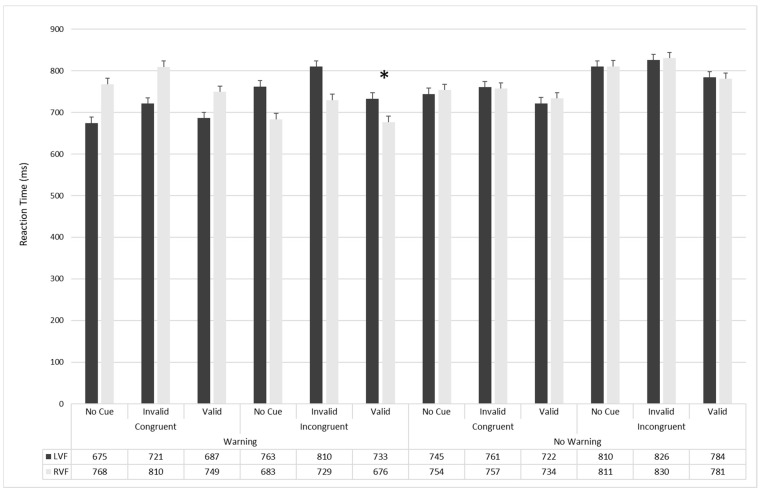
Main reaction times (in milliseconds) for each condition of the LANTI-F, separately for the LVF and the RVF. *: *p* < 0.05.

The *Cue × Flanker* (F_2336_ = 5.71; *p* < 0.005; η^2^ = 0.03), the Cue × Warning (F_2336_ = 19.91; *p* < 0.0001; η^2^ = 0.11), and the Flanker × Warning (F_1168_ = 15.66; *p* < 0.0001; η^2^ = 0.08) interactions were significant. The Visual Field × Warning × Flanker × Cue interaction was also significant (F_2336_ = 4.47; *p* < 0.01; η^2^ = 0.03): the participants were faster in the warning condition, when the flanker was incongruent and the cue was valid for the stimulus presented in the left visual field compared to in the right visual field (733 ± 7.92 ms; 95% CI = 718–749 vs. 749 ± 8.91 ms; 95% CI = 732–767; F_1168_ = 7.2; *p* < 0.01; η^2^ = 0.88; Figure 2). 

The Age Groups × Cue interaction was significant (F_4336_ = 3.16; *p* < 0.01; η^2^ = 0.03) and showed that each group was faster in the valid condition compared to both the invalid condition (Youth: F_1168_ = 59.9, *p* < 0.0001, η^2^ = 0.98; Adults: F_1168_ = 56.6, *p* < 0.0001, η^2^ = 0.98; Elderly/Older: F_1168_ = 133.3, *p* < 0.0001, η^2^= 0.99) and the no-cue condition(Youth: F_1,168_ = 13.7, *p* < 0.002, η^2^ = 0.93; Adults: F_1168_ = 6.8, *p* < 0.01, η^2^ = 0.87; Elderly/Older: F_1168_ = 28.5, *p* < 0.0001, η^2^ = 0.97). Each group was also faster in no cue condition, compared to in the invalid one (Youth: F_1168_ = 25.88, *p* < 0.0001, η^2^ = 0.96; Adults: F_1168_ = 34.7, *p* < 0.0001, η^2^ = 0.97; Elderly/Older: F_1168_ = 60.3, *p* < 0.0001, η^2^ = 0.98). Moreover, Elderly/Older had a greater validity effect (invalid trials-valid trials) (880 ± 38.2 ms; 95% CI = 805–956 vs. 820 ± 37.3 ms; 95% CI = 746–893; F_1168_ = 133.3; *p* < 0.0001; η^2^ = 0.99) than both the Youth group (665 ± 38.2 ms; 95% CI = 590–740 vs. 624 ± 37.3 ms; 95% CI = 551–698; F_1168_ = 59.9; *p* < 0.0001; η^2^ = 0.98) and the Adults group (795 ± 38.2 ms; 95% CI = 720–870 vs. 755 ± 37.3 ms; 95% CI = 682–829 ms; F_1168_ = 56.6; *p* < 0.0001; η^2^= 0.98).

The *Age Groups* × *Flanker type* (F_2168_ = 2.79; *p* = 0.06), the Age Groups × Warning (F_2168_ = 0.30; *p* = 0.74; η^2^= 0.003), and Age Groups × Visual Field (F_2168_ = 0.84; *p* = 0.43; η^2^ = 0.009) interactions were not significant.

**Figure 3:**
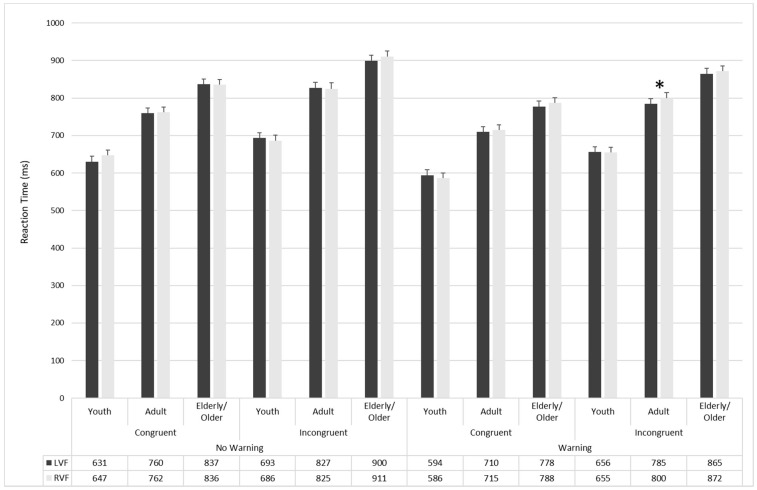
Main reaction times (in milliseconds) for the three age groups in the LVF and RVF as a function of warning and flanker types. *: *p* < 0.05.

The Age Groups × Visual Field × Warning × Flanker interaction was not significant (F_2336_ = 3.14; *p* < 0.05; η^2^ = 0.05). However, planned comparisons revealed that the participants in the Youth group were faster in the no-warning condition when a congruent-flanker trial was presented in the left visual field than in the right visual field (630 ± 24.3 ms; 95% CI = 582–678 vs. 647 ± 23.7 ms; 95% CI = 600–694; F_1168_ = 5.79; *p* < 0.01; η^2^ = 0.85), while the adults’ group was faster in the warning condition when an incongruent flanker trial was presented in the left visual field than in the right visual field (784 ± 24.6 ms; 95% CI = 736–833 vs. 800 ± 24.6 ms; 95% CI = 751–848; F_1168_ = 4.94; *p* < 0.02; η^2^ = 0.83). There were no hemispherical differences in the Elderly/Older group (see Figure 3).

### 3.2. Attentional Effects

To evaluate the age-related changes in the hemispheric lateralization of attentional effects, a preliminary correlational analysis was conducted (Table 3), showing that age correlated with alerting effects in the LVF (r = 0.20; *p* < 0.01) and with conflict effects in the RVF (r = 0.16; *p* < 0.05; Table 3).

To examine the latent role of age, a general linear model considering Age as a continuous predictor and Visual Field and Attentional Effects (Alerting; Conflict and Orienting) as dependent within variables was conducted. A main effect of Age (F_1169_ = 4.29; *p* = 0.04; η^2^ = 0.025) emerged (see Table 4). The main effects of Visual Field (F_1169_ = 3.06; *p* = 0.08; η^2^ = 0.018) and Attentional Effects (F_2338_ = 1.34; *p* = 0.26; η^2^ = 0.008) were not significant. The interaction between Visual Field × Age (F_1169_ = 1.73; *p* = 0.19; η^2^ = 0.010) and *Attentional* Effects × Age (F_1169_ < 1; *p* = 0.88; η^2^ = 0.001) were not significant. Visual Field × Attentional Effects (F_2338_ = 7.72; *p* < 0.001; η^2^ = 0.04) and Age × Visual Field × Attentional Effects (F_2338_ = 5.19; *p* < 0.01; η^2^ = 0.03) interactions were significant.

**Figure 4:**
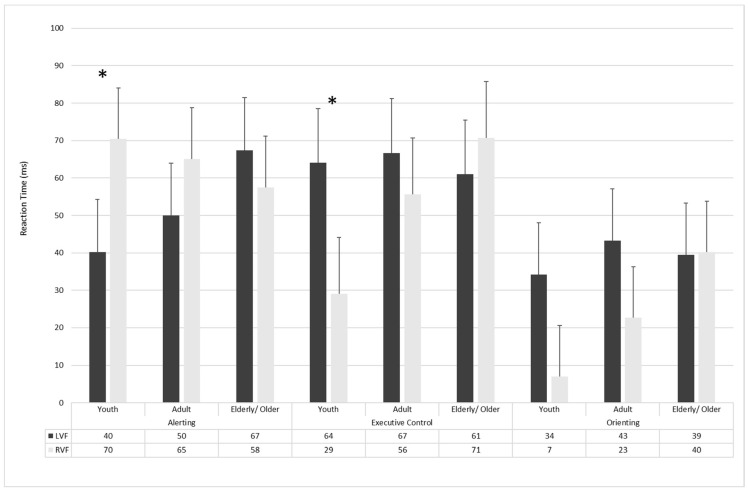
The alerting, conflict, and orienting effects as a function of visual field and age. *: *p* < 0.05.

To further analyze the hemispheric lateralization of attentional effects, an *Age* (Youth, Adults, and Elderly/Older) *×* Visual Field (Left and Right) ANOVAs were conducted on alerting, conflict, and orienting effects (see Figure 4).

Alerting effect: The Age × Visual Field was significant (F_2168_ = 3.40; *p* = 0.04; η^2^ = 0.039). The planned comparisons revealed that alerting effect was lateralized in the Youth group (F_1168_ = 7.54; *p* = 0.007; η^2^ = 0.88), but not in Adults (F_1168_ = 1.89; *p* = 0.17; η^2^ = 0.65) and Elderly/Older (F_1168_ = 0.82; *p* = 0.37; η^2^ = 0.45) groups.

Conflict effect: The Age × Visual Field was significant (F_2168_ = 3.85; *p* = 0.02; η^2^ = 0.04). The planned comparisons revealed that conflict effect was lateralized in the Youth group (F_1168_ = 9.45; *p* = 0.002; η^2^ = 0.90), but not in Adults (F_1168_ = 0.94; *p* = 0.33; η^2^ = 0.48) and Elderly/Older (F_1168_ = 0.73; *p* = 0.39; η^2^ = 0.42) groups.

Orienting effect: The Age × Visual Field was not significant (F_2168_ = 0.77; *p* = 0.46; η^2^ = 0.009).

Finally, to examine the latent role of age, linear regressions were conducted by considering age as an independent variable and alerting, conflict, and orienting effects in both the left and right visual field as the dependent variables (see Table 4). The regression model that considered age as an independent variable revealed a significant effect when alerting in LVF (F_1169_  = 6.50; *p*  <  0.01; R^2^  =  0.04; adjusted R^2^  =  0.03) and conflict in the RVF were considered as dependent variables (F_1169_  = 9.27; *p*  <  0.01; R^2^  =  0.05; adjusted R^2^  =  0.05).

## 4. Discussion

### 4.1. Attentional Networks in Aging

In this study, we analyzed the age-related change in the hemispherical specialization of the attentional networks adopting a lateralized version of the ANTI with non-imperative stimuli (i.e., LANTI-F) [23]

The efficiency and interactions among the three attentional networks (i.e., alerting, orienting, and executive control) were confirmed (e.g., [23]). As expected, faster reaction times were found in congruent trials, valid cues, warning conditions than in incongruent trials, invalid cues, and no-warning conditions, respectively. These results confirmed a preserved functionality of the three attentional systems from youth to old age [32]. Nevertheless, an age-related generalized slowness of RTs confirms also a general decline of attentional processes (e.g., [32,70]).

This study revealed a higher conflict effect in the older group, proving a worse executive functioning. Accordingly, conflict control seems to present a linear pattern characterized by a decline during aging, supporting previous evidence that adopted both imperative (i.e., arrow; [33,35,36,37,38] or non-imperative stimuli (i.e., faces [32]). 

Regarding the orienting network, a linear decrease was found. Our results revealed significantly slower reaction times in the elderly than in the younger groups in invalid trials, confirming that older people seem to be unable to disengage from an invalid spatial cue and re-orienting attention to an unexpected location. This effect could be due to the difficulty of shifting attention to a new location or to a greater cost in disengaging [55,71,72]. It is also consistent with the decline of the executive system, highlighted by the conflict effect.

Unexpectedly, the presentation of an alerting stimulus seems not to affect the performance in aging. This result contrasts with previous studies that identified either an improvement [34] or a worsening [33,35,36,37,38] in the performances of older people associated with alerting stimulus. This difference could be due to the characteristics of the warning signal. In the studies mentioned above, the warning signal was a visual stimulus, while we adopted an auditory stimulus. Although the acoustic signal, as the visual one, induces a phasic increase of alertness [73], some authors suggested that the auditory modality might generate alertness more automatically than the visual modality [74]. For this reason, the LANTI-F may have helped the participants indiscriminately, flattening the differences due to age. This effect could have been due to the greater facility of the LANTI-F than those of the other versions of the ANT due to non-directional colored stimuli being easier to discriminate [23].

### 4.2. Hemispheric Specialization of Attentional Networks in Aging

Concerning hemispheric lateralization, the participants tended to respond faster to stimuli presented in the left visual field than those presented in the right one, supporting the dominance of the right contralateral hemisphere in attention [22,75]. Moreover, the results confirm the age-related changes in the hemispheric specialization of attention observed by other authors [64,65].

The Youth and Adults groups seemed to resolve the congruent or incongruent conditions presented in the left visual field faster. However, only the Adults group had an advantage due to the warning signal, supporting the hypothesis that increased alertness improves the ability to respond to stimuli presented in the left visual field [76]. Correlational and regression analyses confirmed the age-related changes in the alerting effects, showing that alerting signals improve performance with increasing age, but only in the right hemisphere. While the presence of a hemispheric asymmetry in young and adult participants considering the Warning × Flanker interactions could support a right hemisphere dominance in the attentional process [52,53]; the absence of lateralization of the attentional network in the elderly would support the hypothesis that the structure and organization of the brain change during healthy aging [60,61,62]. 

The decreased hemispheric asymmetry in the elderly can be due to an age-related compensatory process aimed to contrast the physiological decline. Although the younger showed a dominance of the right hemisphere for the alerting system, the older group presented an opposite pattern. According to other authors, we suggest that this result could be explained by the involvement of the analog area of the intact hemisphere to preserve the attentional processes [64,77,78].

Our findings could be explained according to the right hemi-aging model (RHAM), which hypotheses a faster decline in the right hemisphere than in the left one [79]. The lower right hemisphere specialization in the elderly would seem to agree with the preserved performance in language tasks (i.e., in the left hemisphere) [80]. Moreover, in line with the hemispheric asymmetry reduction in older adults (HAROLD), this hemispheric asymmetry could be due to a compensatory or a dedifferentiation process [77,78]. Accordingly, the functions related to the right hemisphere deterioration could be recovered thanks to the involvement of the analog area of the preserved left hemisphere, leading to a lower hemispheric specialization and a more bilateral pattern of hemispheric functioning.

Furthermore, the LANTI-F could not be as easier for the elderly as the younger group. Some studies [69] reported task-related hemispheric lateralization: increased task complexity and cognitive load would determine bi-hemispheric processing in the elderly, for whom the unilateral processing, characterizing younger people, would be insufficient to solve a harder task.

Despite the reported evidence, this study is characterized by some limitations. Firstly, the absence of neurophysiological measures (e.g., EEG; fMRI) precludes us from determining the causal effect between the attentional networks and the neural mechanisms. Moreover, comparing responses from the three age groups to tasks with imperative (i.e., LANTI-arrow) and non-imperative (i.e., LANTI-F) stimuli would have been helpful to determine whether the task complexity could play a role in evidencing different hemispheric lateralization in healthy aging. 

Future studies could explore the hemispheric specialization in healthy aging and the pathological one, as Alzheimer’s disease or mild cognitive impairment, in which both executive functioning and attentional processes are compromised [70,81]. 

The evidence of this study shows that the lateralization of attentional processes varies in aging. Aging would appear to reduce hemispheric specialization related to attentional processes; future studies could help identify if task complexity could confirm or extend our results.

## Figures and Tables

**Table 1:** Means and standard deviations of age, year of education, Mini-Mental State Examination (MMSE), and intelligence quotient (IQ) for each group of participants.

Group	Age	Years of Education	MMSE	IQ
Youth	23.46 ± 2.08	16.39 ± 1.53	27.66 ± 0.74	114.30 ± 13.06
Adults	55.26 ±7.54	14.35 ± 4.30	27.85 ± 1.34	118.65 ± 10.09
Elderly/Older	71.82 ± 5.88	13.16 ± 5.11	26.80 ± 1.55	113.37 ± 13.37

**Table 2:** Mean reaction times (in ms) and standard deviations of the three age groups of participants for each condition and each visual field.

			Youth		Adults		Elderly/Older	
			RVF	LVF	RVF	LVF	RVF	LVF
No-Warning	Congruent	Valid	643.04 17.9	610.9 16.15	742.89 17.9	736.58 16.15	815.36 17.9	817.95 16.15
		Invalid	650.03 15.04	645.13 14.79	765.56 15.04	779.87 14.79	855.51 15.04	857.44 14.8
		No-cue	648.64 14.86	635.56 15.57	776.61 14.86	763.4 15.57	838.16 14.8	834.87 15.57
	Incongruent	Valid	656.06 16.02	668.05 15.71	803.84 16.02	809.71 15.71	882.91 16.01	875.07 15.71
		Invalid	708.5 16.85	705.71 16.51	844.68 16.85	847.53 16.52	938.19 16.85	924.01 16.52
		No-cue	693.67 17.65	706.66 15.98	826.95 17.65	823.68 15.99	912.13 17.65	899.79 15.99
Warning	Congruent	Valid	573.28 15.07	585.23 16.06	696.09 15.07	713.58 16.05	759.4 15.07	761.34 16.05
		Invalid	608.13 14.88	617.36 15.22	754.77 14.88	730.55 15.23	824.2 14.8	815.33 15.23
		No-cue	577.61 16.39	580.80 15.17	693.28 16.39	686.44 15.17	779.1 16.39	756.6 15.17
	Incongruent	Valid	633.73 15.44	624 13.71	782.53 15.44	760.05 13.71	831.46 15.44	814.86 13.71
		Invalid	689.98 16.6	697.49 16.48	825.7 16.6	814.42 16.48	913.7 16.6	917.88 16.48
		No-cue	641.57 16.05	645 16.88	791.2 16.05	779.29 16.88	870.44 16.05	862.25 16.88

LVF: left visual field; RVF: right visual field.

**Table 3:** Partial correlations between age and the three attentional effects for the LVF and RVF (confounding variables: years of education; MMSE; IQ).

			Left Visual Field			Right Visual Field		
		Age	Alerting	Conflict	Orienting	Alerting	Conflict	Orienting
**Left Visual Field**	**Alerting**	0.20 **	-					
	**Conflict**	−0.05	−0.40 **	-				
	**Orienting**	0.01	−0.14	0.31 **	-			
**Right Visual Field**	**Alerting**	–0.04	0.15	−0.01	−0.01	-		
	**Conflict**	0.16 *	−0.05	0.30 **	0.09	−0.22 *	-	
	**Orienting**	0.12	0.06	0.14	0.15 *	−0.30 **	0.21 **	-

* *p* < 0.05; ** *p* < 0.01.

**Table 4:** Regression analysis considering age as an independent variable and attentional effects in the LVF and RVF as dependent variables.

		Model	*B*	Standard Error	Beta	*t*	Sign (*p*)	95% CI Lower	95% CI Upper
LVF	Alerting	Age	**0.58**	**0.23**	**0.19**	**2.55**	**0.01**	**0.13**	**1.03**
	Conflict	Age	−0.12	0.26	−0.03	−0.46	−65	−0.63	0.39
	Orienting	Age	−0.04	0.31	−0.01	−0.12	0.91	−0.64	0.57
RVF	Alerting	Age	−0.16	0.24	−0.51	−0.66	0.51	−0.64	0.32
	Conflict	Age	**0.84**	**0.27**	**0.23**	**3.04**	**0.003**	**0.29**	**1.38**
	Orienting	Age	0.48	0.40	0.09	1.20	0.23	−0.31	1.27

## Data Availability

Data supporting reported results can be required to corresponding author.
